# A bioinformatics analysis to identify novel biomarkers for prognosis of pulmonary tuberculosis

**DOI:** 10.1186/s12890-020-01316-2

**Published:** 2020-10-24

**Authors:** Yahong Sun, Gang Chen, Zhihao Liu, Lina Yu, Yan Shang

**Affiliations:** 1 Department of Pulmonary and Critical Care Medicine, Haining People’s Hospital, Jiaxing, 314400 China; 2 Department of Respiratory and Critical Care Medicine, Changhai Hospital, Naval Medical University (Second Military Medical University), No. 168 Changhai Road, Yangpu District, Shanghai, 200433 China

**Keywords:** Pulmonary tuberculosis, Clustering analysis, Enrichment analysis, Hub gene, PPI network

## Abstract

**Background:**

Due to the fact that pulmonary tuberculosis (PTB) is a highly infectious respiratory disease characterized by high herd susceptibility and hard to be treated, this study aimed to search novel effective biomarkers to improve the prognosis and treatment of PTB patients.

**Methods:**

Firstly, bioinformatics analysis was performed to identify PTB-related differentially expressed genes (DEGs) from GEO database, which were then subjected to GO annotation and KEGG pathway enrichment analysis to initially describe their functions. Afterwards, clustering analysis was conducted to identify PTB-related gene clusters and relevant PPI networks were established using the STRING database.

**Results:**

Based on the further differential and clustering analyses, 10 DEGs decreased during PTB development were identified and considered as candidate hub genes. Besides, we retrospectively analyzed some relevant studies and found that 7 genes (CCL20, PTGS2, ICAM1, TIMP1, MMP9, CXCL8 and IL6) presented an intimate correlation with PTB development and had the potential serving as biomarkers.

**Conclusions:**

Overall, this study provides a theoretical basis for research on novel biomarkers of PTB, and helps to estimate PTB prognosis as well as probe into targeted molecular treatment.

**Supplementary information:**

**Supplementary information** accompanies this paper at 10.1186/s12890-020-01316-2.

## Background

Tuberculosis (TB) is a kind of chronic infectious disease induced by *Mycobacterium tuberculosis* (MTB) with a relatively high rate of morbidity and mortality, and it has developed as a threatening public health issue globally (www.who.int/tb/publications/global_report/en/). According to the statistics reported by the World Health Organization in 2019, there were approximately 10 million newly diagnosed TB cases and about 1.4 million deaths worldwide (including HIV-positive people), and the top death toll was observed in low- and middle-income countries (http://apps.who.int/iris). Pulmonary tuberculous (PTB) is the most common TB form [1], and the prevention of PTB-related death can be greatly achieved via early effective diagnosis [2]. Therefore, mining potential biomarkers associated with PTB occurrence and development is vital for PTB early diagnosis, prognosis assessment and individualized treatment.

Clinically, disease-related biomarkers that are able to predict possible responses before the start of treatment or monitor follow-up therapeutic responses are crucial for PTB treatment, as they can potentially identify the patients with a big bacterial load and/or enhanced inflammatory response, which allows doctors to provide more intensive surveillance and effective therapeutic strategies of a long period [3]. As an alternative of sputum examination, serum-based biomarkers have attracted much attention in recent years. Unlike sputum, serum is relatively easy to be collected and it remains the available source of biomarkers during treatment. Besides, serum-derived inflammatory and infectious markers are quantified, and multiple biomarkers can be combined into a predictive biomarker signature, which can greatly increase the predictive accuracy [4–7]. Recently, some biomarkers have been verified to be implicated in PTB occurrence and development, and can be used for PTB prognosis in clinic. For instance, Klassert TE et al. [8] found that serum *MASP1* was significantly increased in PTB patients thus affecting the lectin pathway complement activity in vitro, and it could be involved in PTB occurrence under the MTB pathogenesis. In addition, Yuzo Suzuki et al. [9] also discovered elevated *sCD206* in serum of PTB patients, which presented a close relationship with prognosis and had been recognized as a potential biomarker. Nevertheless, there is still a need for effective biomarkers related to PTB development [2], which is of great significance for PTB control globally.

This study applied bioinformatics analysis on the gene expression profiles of PTB in GEO database and identified PTB-related hub genes via clustering analysis and PPI networks. In the meantime, these hub genes were analyzed for their functions in as well as associations with PTB occurrence and development, which in turn helps to exploit the potential genes valuable for PTB treatment and prognosis estimation.

## Methods

### Data collection

Expression matrix relevant to PTB was accessed from the GEO database. The enrolled expression microarray was in accordance with the criterion that healthy controls, TB samples and post-treatment samples (*n* ≥ 30) shall be included. GSE54992 microarray was eventually screened for this study, comprising 39 samples in total classified as HC (healthy controls, *n* = 6), LTBI (latent tuberculosis infection, *n =* 6), TB/TB0 (tuberculosis/ 0 month after initiation of anti-TB chemotherapy, *n* = 9), TB3 (3 months after initiation of anti-TB chemotherapy, *n =* 9) and TB6 (6 months after initiation of anti-TB chemotherapy, *n =* 9).

### Data processing

Firstly, the expression data of the GSE54992 microarray were treated by the KNN algorithm of R language and then normalized. The “limma” package was used to perform differential analysis on the normalized data to identify the differentially expressed genes (DEGs) in the cases of TB vs LTBI and TB vs HC, with the threshold set as |log2FC| > 1.5 and FDR < 0.05. The overlapping DEGs were identified for subsequent analysis.

### Enrichment analysis on the overlapping DEGs

Gene Ontology (GO) and Kyoto Encyclopedia of Genes and Genomes (KEGG) enrichment analyses were performed on the overlapping DEGs using the “ClusterProfiler” package. Based on the GO analysis, gene annotations were applied to describe the biological role of a gene product in regard to three aspects: molecular function (MF), biological process (BP) and cellular component (CC). FDR < 0.05 was set as the threshold.

### Clustering analysis

TCseq package is a tool that can be used to analyze different types of time course sequencing data via providing a unified suite [10]. In this study, the TCseq package was employed to classify the overlapping DEGs into various types of Clusters (K = 6), with the genes in each Cluster were then processed for GO annotation and KEGG enrichment analysis.

### Protein-protein interaction (PPI) network construction

The Search Tool for the Retrieval of Interacting Genes/Proteins database (STRING; https://string-db.org/) is a public database harboring known and predicted protein-protein interactions [11]. Protein-protein interaction (PPI) is an indispensable approach for research on protein functions as it helps to clarify the interactions among proteins. In this study, the STRING database was used to construct a PPI network with an interaction score > 0.4. The network was then visualized using the Cytoscape software (version 3.7.0).

## Results

### Identification of DEGs in PTB

**Fig. 1:**
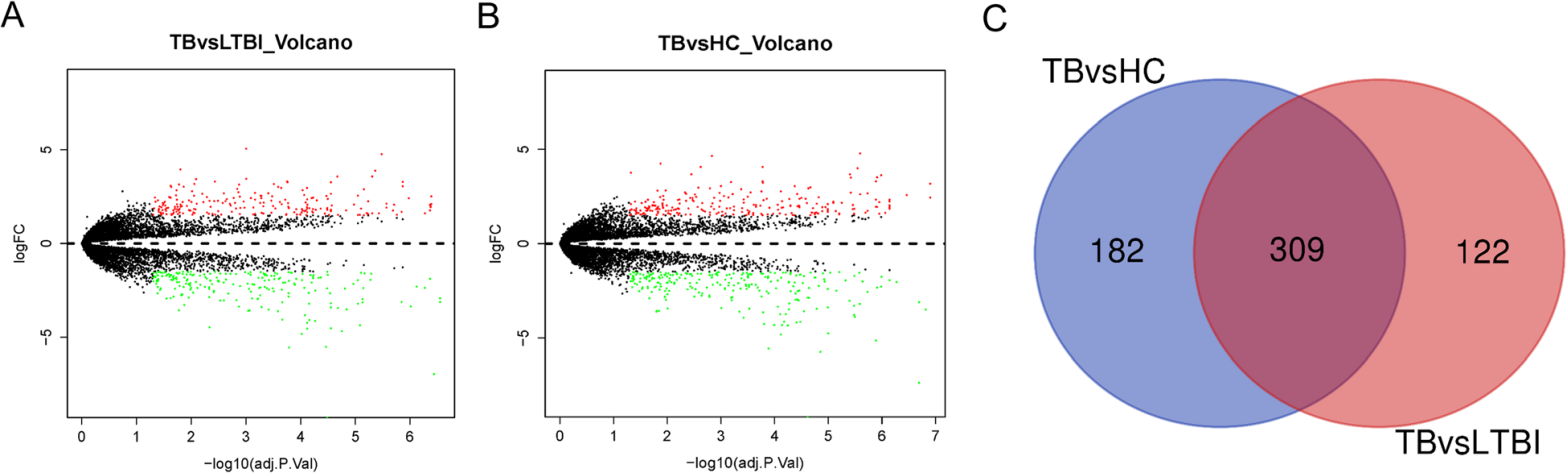
Identification of DEGs in PTB. **a**, **b**: Volcano plots were made to screen the DEGs from TB patients compared to LTBI or HC. Black dots represent genes that are not differentially expressed between TB patients and LTBI or HC, whereas the green dots and red dots represent the down-regulated and up-regulated genes, respectively; **c**: A Venn Diagram was drawn for identifying the overlapping DEGs among TB vs HC vs LTBI

Differential analysis was performed on the gene expression data from the PTB microarray GSE54992. In all, 431 DEGs in TB vs LTBI (including 212 up-regulated genes and 219 down-regulated genes) and 491 DEGs in TB vs HC (including 241 up-regulated genes and 250 down-regulated genes) were identified as shown in Fig. 1a and b. Besides, a Venn Diagram was plotted and 309 overlapping DEGs were identified (Fig. 1c), which were used for follow-up analysis.

### Enrichment analysis on the overlapping DEGs

**Fig. 2:**
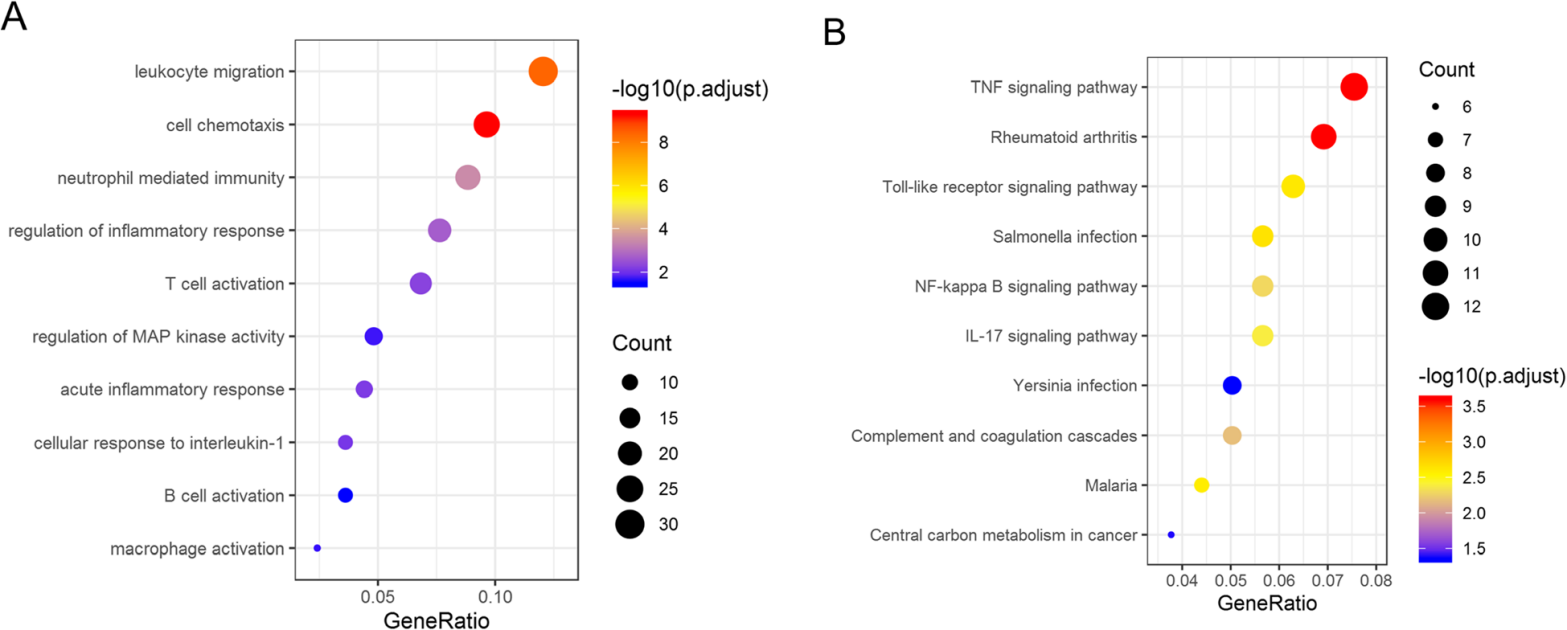
GO and KEGG enrichment analyses on the overlapping DEGs. **a**: The most enriched GO terms of the DEGs; **b**: The most enriched KEGG pathways of the DEGs

GO and KEGG enrichment analyses were conducted to explore the biological function of the 309 overlapping DEGs. Based on the GO analysis, these DEGs were mainly activated in inflammation- and immunoregulation-associated functions, as indicated by the top 10 most enriched biological activities containing leukocyte migration, cell chemotaxis, neutrophil mediated immunity, regulation of inflammatory response, T cell activation, regulation of MAP kinase activity, acute inflammatory response, cellular response to interleukin-1, B cell activation and macrophage activation (Fig. 2a). In addition, KEGG analysis suggested that these DEGs were predominantly enriched in NF-kappa B signaling pathway, TNF signaling pathway, Toll-like receptor signaling pathway, IL-17 signaling pathway, complement and coagulation cascades and other pathways intimately relevant to inflammation and immune (Fig. 2b). These results collectively demonstrated that the 309 overlapping DEGs exerted their roles predominantly in inflammatory and immunoregulatory processes during PTB occurrence and development.

### Clustering analysis and further enrichment analysis

**Fig. 3:**
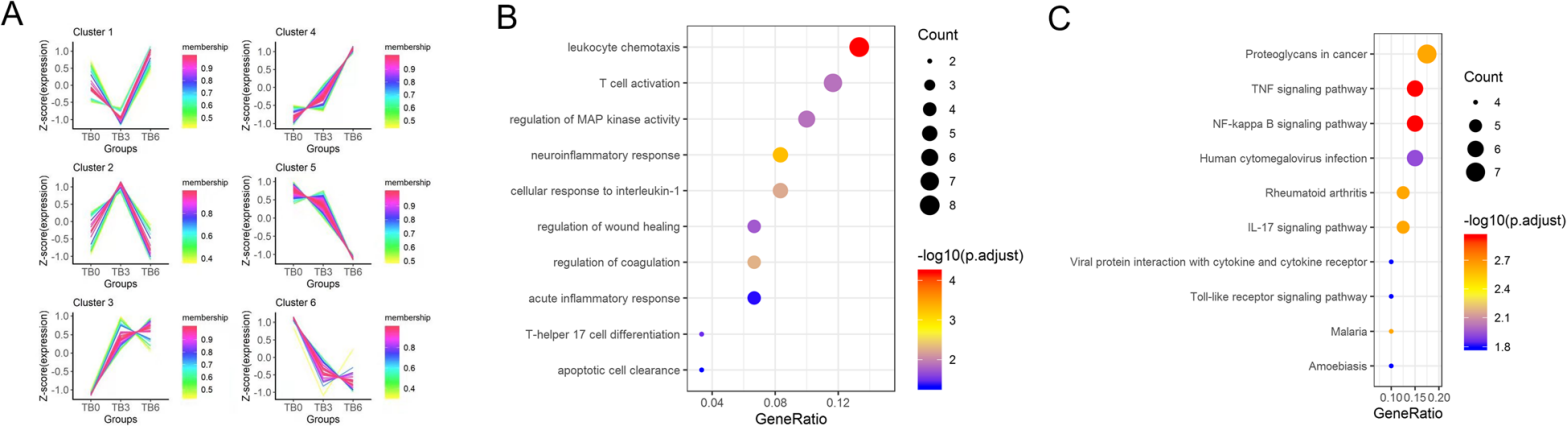
Clustering analysis and enrichment analysis. **a**: Clustering analysis was performed to find gene Clusters in anti-TB chemotherapy-treated samples. All overlapping DEGs were divided into several categories according to their expression levels. The Abscissa is the Cluster, and the ordinate is the corrected Z-score of the expression. The larger the corrected Z-score, the higher the expression level, and vice versa, the lower the expression level. Each broken line represents a gene. The greater the value the color represents, the closer the gene is to the average level in the classification; **b**: The most enriched GO terms of the DEGs in the Cluster 4; **c**: The most enriched KEGG pathways of the DEGs in the Cluster 4

After a preliminary understanding of the biological functions of the overlapping DEGs, clustering analysis was conducted for in-depth research. As revealed in Fig. 3a, these DEGs were clustered into 6 Clusters. In anti-TB chemotherapy-treated samples, the level of the genes in Cluster 1 was decreased firstly and increased afterwards and the minimum level appeared at the third month, whereas the level of the genes in Cluster 2 exhibited an opposite expression trend. Besides, the level of the genes in Cluster 3 and Cluster 4 were elevated with time going by. Reversely, the expression level of the genes in Cluster 5 and Cluster 6 were declined with time going by. Thereafter, GO and KEGG enrichment analyses were performed, finding that there was no result satisfied considering the genes in Cluster 1, 2 and 6, while only genes in Cluster 4 presented an intimate correlation with PTB. KEGG analysis discovered that the genes in Cluster 4 were mainly enriched in NF-kappa B signaling pathway, TNF signaling pathway, Toll-like receptor signaling pathway, IL-17 signaling pathway and other immune-related pathways, and GO analysis showed some major immune functions, such as T cell activation, apoptotic cell clearance, leukocyte chemotaxis and acute inflammatory response (Fig. 3b and c). Genes in Cluster 4 were thereby selected for further analysis.

### PPI network construction and hub gene identification

**Fig. 4:**
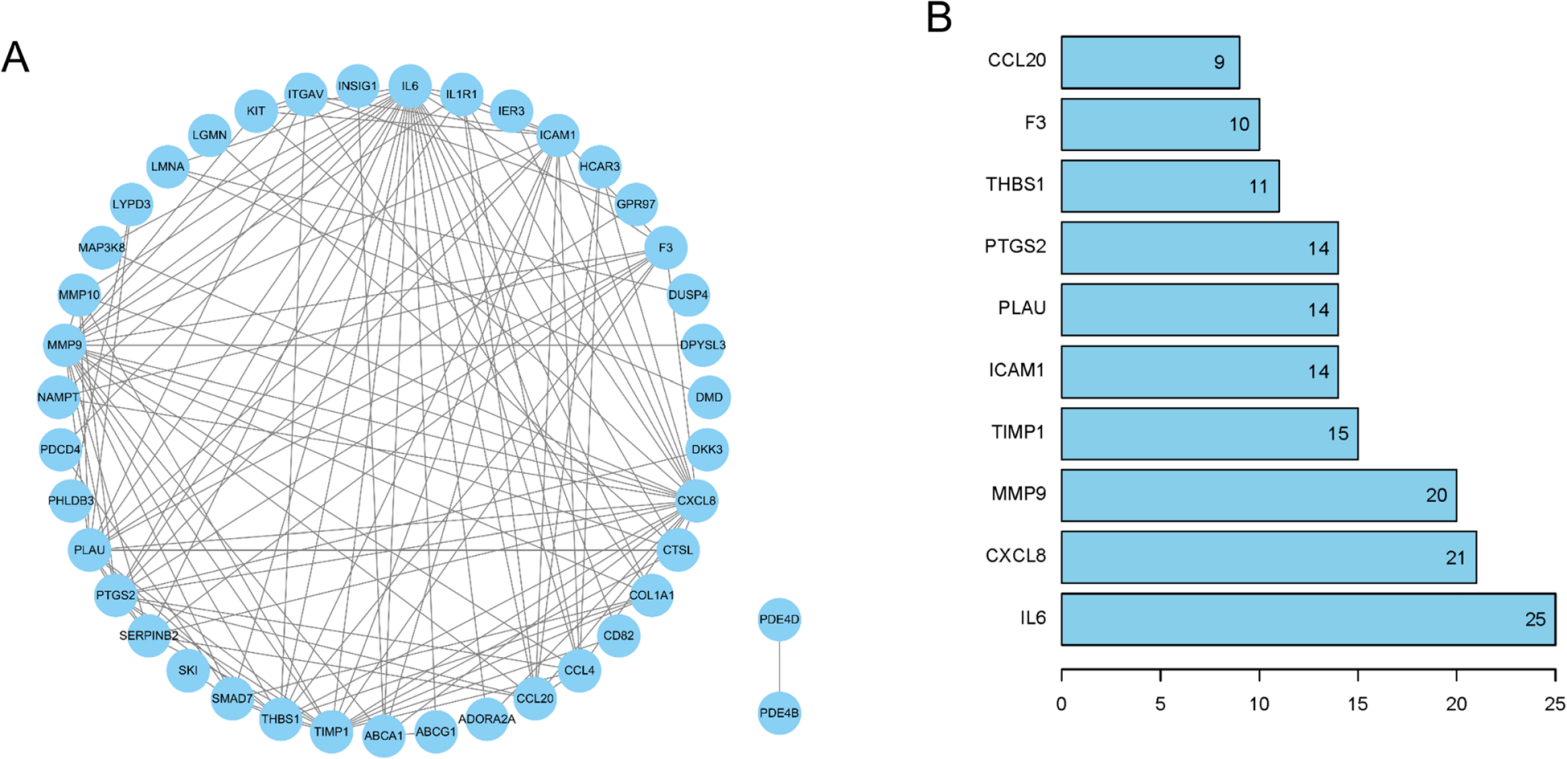
PPI network construction and hub genes identification. **a**: The PPI network based on the genes in the Cluster 4; **b**: The top 10 genes with a relatively high node degree

DEGs in Cluster 4 were projected onto a STRING network for functional enrichment analysis. A PPI network bearing totally 39 nodes were sequentially established with the threshold set as interaction score > 0.4 (Fig. 4a). Besides, the top 10 genes with a relatively high node degree were defined as hub genes and listed in Fig. 4b. Differential and clustering analyses showed that these hub genes were all down-regulated during PTB development (detailed in Supplementary Table), and then up-regulated after patients underwent anti-TB chemotherapy. In view of these, we reasoned that the top 10 genes might play an inhibitory role in PTB progression.

## Discussion

It has been reported that great progress has been made on the effective epidemic control of PTB due to the implement of the National TB Control Programme (2011–2015). However, despite the reduction in prevalence of smear-positive PTB cases (170/100,000 vs 57/100,000), the burden of drug-resistant PTB is still sizable, which prompts us to explore effective biomarkers for the improvement of current PTB treatment [12, 13]. Currently, there have been studies on identifying PTB-related biomarkers for early diagnosis or prognosis estimation. For instance, Guanren et al. [14] used bioinformatics analysis combined with clinical biochemical examination and found that the gene expression and protein content of serum SLAMF8, LILRB4 and IL-10Ra were all significantly elevated in PTB patients, and all these three genes were associated with poor prognosis. Michael et al. [15] identified 10 metabolites of MTB from the volatile organic compounds (VOCs) in breath, which were remarkably increased and could be used as biomarkers for PTB diagnosis. This study adopted bioinformatics methods to identify DEGs in PTB from the GEO database, which were then processed for clustering analysis and projected into a PPI network for screening candidate hub genes (CCL20, F3, THBS1, PTGS2, PLAU, ICAM1, TIMP1, MMP9, CXCL8 and IL6) that were intimately associated with PTB occurrence and development. Hence, to clarify whether these hub genes have the potential serving as biomarkers of PTB, we retrospectively analyzed relevant research on PTB.

C-C motif chemokine ligand 20 (CCL20) is a special chemokine ligand of the C-C motif chemokine receptor 6 (CCR6) functioning under multiple pathological conditions [16]. It’s reported that cytokines and chemokines both participate in protective immunity and immunopathogenesis of TB, as well as in MTB-host-pathogen interactions [17]. Lee JS et al. [18] investigated the level of CCL20 and the corresponding regulatory mechanism in PTB cases and healthy controls, finding that CCL20 was up-regulated in PTB patients and mediated by proinflammatory cytokines. PTGS2 (Prostaglandin-endoperoxide synthase 2), also known as cyclooxygenase-2 (COX-2), is a type of enzyme responsible for generation of intermediate PGH. For TB-infectious macrophages, PGH-induced repair for plasma membrane damage is crucial [19]. Moreover, the mechanism by which MTB regulates COX-2 expression in macrophages is reported to be an important factor during the initiation or maintenance of host immune response [20]. Wang L et al. [21] revealed that COX-2 inhibition could suppress the apoptosis of macrophages induced by secreted MTB lipoprotein. Rand L et al. [22] reported that COX-2 could inhibit p38MAPK-PG signaling pathway to decrease *MMP-1* activity, which could be considered as a therapeutic target to attenuate the damage of PTB inflammatory tissues. *ICAM1* (Intercellular adhesion molecule 1; *CD54*), a member of immunoglobulin super family (Igsf) [23], is necessary for cell adhesion and acts as an important player in inflammation-induced tissue adhesion, tumor metastasis and immune response [24]. Du SS et al. [25] identified some differentially expressed proteins associated with PTB diagnosis using protein microarray technique, and found that *ICAM1* had relatively high sensitivity and specificity and had the potential serving as an indicator for sputum-negative PTB diagnosis. *MMP-9* has been discovered to be involved in the recruitment of macrophages and granuloma occurrence as suggested by Jennifer L et al., and early *MMP* activity is a crucial part for lung MTB infection resistance. To be specific, *MMP-9* is a necessity for macrophage recruitment and tissue remodeling during PTB progression [26]. CXCL8 (C-X-C motif chemokine ligand 8) inflammatory cytokine can be released during the activation of macrophages so as to foster the establishment of immune system network, and it has been detected to be up-regulated in PTB sufferers [27]. Block DC et al. [28] described that CXCL8 was the natural immune regulator in active PTB patients. IL6 (interleukin 6) is regarded to be a biomarker for predicting the death of HIV-negative PTB patients as supported by Wang Q et al. [29] Besides, IL6 is also believed to be associated with MTB infection and PTB susceptibility [30]. Similarly, the alteration of fibrosis-related TIMP1 has been identified to be tightly relevant to the pathological basis of PTB susceptibility, as revealed by Marquis JF et al. [31]. Collectively, the above results demonstrate that these hub genes can function during PTB occurrence and development by serving as immune regulators, therapeutic targets, and potential biomarkers, and they can affect PTB susceptibility and resist MTB infection. In addition, these results support our study on mining effective biomarkers of PTB from the 10 candidate hub genes. Furthermore, some other genes like F3, THBS1 and PLAU have not been investigated currently for their role in improvement of PTB treatment.

Although a relatively accurate prediction for PTB prognosis could be achieved by the above hub genes we identified, there are still some limitations in this study. TB is a multifactorial disease that can be divided into non-tuberculous mycobacteria (NTM) infections and MTB based on the type of pathogen. NTM infections are predominantly caused by mycobacteria except *Mycobacterium tuberculosis*, Mycobacterium bovis and Mycobacterium leprae, with symptoms similar to MTB, making it hard to be diagnosed in clinic. Besides, NTM infections are less toxic relative to MTB but have similar clinical manifestations to MTB, and the identification of NTM infections is generally realized by means of bacterial culture [32]. Studies believed that patients have various physiological and biochemical responses to NTM infections and MTB. Feng et al. [33] made a study on macrophages and believed that the activation of NF-κB in MTB patients was more significant in comparison with that in patients with NTM infections, and there were differences in IL-8, IL-10 and TNF-α in different infections. Additionally, Nurlela et al. [34] also discovered that level of TNF-α in pleural fluid of patients with NTM infections and MTB was different, with that in MTB sufferers significantly higher. In the present study, due to the lack of proper data, analysis for the TB patients infected by different pathogens was not conducted. Besides, this study is purely a bioinformatics analysis without any in vivo and in vitro data. Therefore, more analyses should be carried out to help us gain more insight into the 10 hub genes, so as to bring benefit to the patients with TB.

## Conclusion

In sum, based on a series of bioinformatics methods and a retrospective analysis, our study identified 7 hub genes which showed an intimate correlation with PTB development and prognosis and had the potential acting as therapeutic targets and prognostic indicators. Meanwhile, there are some limitations in our study which will be further solved in our follow-up studies.

## Supplementary information

**Additional file 1.**

## Data Availability

The datasets analysed during the current study are available in the Gene Expression Omnibus repository, https://www.ncbi.nlm.nih.gov/geo/query/acc.cgi?acc=GSE54992.

## Abbreviations

PTB: Pulmonary tuberculosis
DEGs: Differentially expressed genes
TB: Tuberculosis
MTB: *Mycobacterium tuberculosis*
GEO: Gene Expression Omnibus
HC: Healthy controls
LTBI: Latent tuberculosis infection
TB0: 0 month after initiation of anti-TB chemotherapy
TB3: 3 months after initiation of anti-TB chemotherapy
TB6: 6 months after initiation of anti-TB chemotherapy
GO: Gene Ontology
KEGG: Kyoto Encyclopedia of Genes and Genomes
MF: Molecular Function
BP: Biological Process
CC: Cellular Component
PPI: Protein-protein interaction
STRING: The Search Tool for the Retrieval of Interacting Genes/Proteins database
VOCs: Volatile organic compounds
CCL20: C-C motif chemokine ligand 20
CCR6: C-C motif chemokine receptor 6
PTGS2: Prostaglandin-endoperoxide synthase 2
COX-2: Cyclooxygenase-2
ICAM1: Intercellular adhesion molecule 1
Igsf: Immunoglobulin super family
CXCL8: C-X-C motif chemokine ligand 8
IL6: Interleukin 6
NTM: Non-tuberculous mycobacteria
